# An Automated Grading System Based on Topological Features for the Evaluation of Corneal Fluorescein Staining in Dry Eye Disease

**DOI:** 10.3390/diagnostics13233533

**Published:** 2023-11-26

**Authors:** Jun Feng, Zi-Kai Ren, Kai-Ni Wang, Hao Guo, Yi-Ran Hao, Yuan-Chao Shu, Lei Tian, Guang-Quan Zhou, Ying Jie

**Affiliations:** 1 Beijing Ophthalmology and Visual Science Key Lab, Beijing Institute of Ophthalmology, Beijing Tongren Eye Center, Beijing Tongren Hospital, Capital Medical University, Beijing 100730, China; 2 School of Biological Science and Medical Engineering, Southeast University, Nanjing 210096, China; 3 College of Control Science and Engineering, Zhejiang University, Hangzhou 310027, China

**Keywords:** dry-eye disease, corneal fluorescein staining, topological features, machine learning

## Abstract

Background: Corneal fluorescein staining is a key biomarker in evaluating dry eye disease. However, subjective scales of corneal fluorescein staining are lacking in consistency and increase the difficulties of an accurate diagnosis for clinicians. This study aimed to propose an automatic machine learning-based method for corneal fluorescein staining evaluation by utilizing prior information about the spatial connection and distribution of the staining region. Methods: We proposed an end-to-end automatic machine learning-based classification model that consists of staining region identification, feature signature construction, and machine learning-based classification, which fully scrutinizes the multiscale topological features together with conventional texture and morphological features. The proposed model was evaluated using retrospective data from Beijing Tongren Hospital. Two masked ophthalmologists scored images independently using the Sjögren’s International Collaborative Clinical Alliance Ocular Staining Score scale. Results: A total of 382 images were enrolled in the study. A signature with six topological features, two textural features, and two morphological features was constructed after feature extraction and selection. Support vector machines showed the best classification performance (accuracy: 82.67%, area under the curve: 96.59%) with the designed signature. Meanwhile, topological features contributed more to the classification, compared with other features. According to the distribution and correlation with features and scores, topological features performed better than others. Conclusions: An automatic machine learning-based method was advanced for corneal fluorescein staining evaluation. The topological features in presenting the spatial connectivity and distribution of staining regions are essential for an efficient corneal fluorescein staining evaluation. This result implies the clinical application of topological features in dry-eye diagnosis and therapeutic effect evaluation.

## 1. Introduction

Dry eye disease (DED) is a multifactorial condition affecting the ocular surface, characterized by a loss of homeostasis of the tear film and accompanied by ocular symptoms, in which ocular surface damage plays an etiological role [1]. The prevalence of DED ranges between 5% and 50% worldwide [2]. To diagnose DED, careful assessment of ocular surface damage is essential. Recommended assessments include corneal, conjunctival, and lid-margin staining, with corneal staining being the most significant [3]. Sodium fluorescein is a common dye for corneal staining, as it can enter sick cells and be transported across altered tight junctions and micro pool at the space left by a shed epithelial cell [4].

Several subjective grading scales have been developed to evaluate corneal fluorescein staining (CFS), including the National Eye Institute (NEI)/Industry scale [5], the Oxford scale [6], and the Sjögren’s International Collaborative Clinical Alliance Ocular Staining Score (OSS) scale [7]. The NEI scale divides the cornea into five zones (central, superior, inferior, nasal, and temporal) and assigns a grade from 0 to 3 to each zone. In contrast, the Oxford and OSS scales examine the entire cornea, using different criteria to evaluate staining regions. The Oxford scale employs illustrative drawings to grade corneal staining from 0 to 5, while the OSS scale counts the number of staining dots and assigns a score between 0 and 3, with an additional point for special types of dot distributions. However, there is little consensus on the features included in these scales, as they differ in their procedures and standards [8]. The variety of grading scales can be confusing for ophthalmologists and time consuming in clinical practice, and subjectivity leads to interobserver and intraobserver variability.

Therefore, computer-aided diagnosis (CAD) has made progress in evaluating CFS in recent years. Rodriguez et al. [9] developed a semi-automatic method by manually identifying staining regions using three seed points in a corneal area. Similarly, Pellegrini et al. [10] manually traced the corneal area with the ImageJ 1.51s software for CFS grading. In contrast, Daugman’s operator was employed for locating the cornea, followed by an Otsu method for automatically identifying staining regions [11]. Bagbaba et al. [12] also developed a method that automatically segmented staining regions using connected component labeling. Kourukmas manually positioned the cornea and segmented the stained area with the help of ImageJ software [13]. Rather than directly predicting the CFS result, all these methods finally attempted to explore the correlation between the corresponding grade and the image-based parameters of the staining regions, such as the number of corneal staining regions and the ratio of the staining area to the total corneal area. Recently, data-driven deep neural networks (DNNs) have been used to segment staining regions or to examine the grading scale directly from the images [14,15,16,17,18], producing promising results in grading CFS.

The emergence of computer-aided solutions for evaluating CFS can provide objective measurements and facilitate the quantitative grading of dry eyes. However, previous studies have primarily focused on the correlation of CFS with the number or area of the staining regions [9,10,11,12,18], neglecting the distribution and texture information from these regions. This approach is inadequate for complicated corneal staining images that involve confluent staining and filaments. Although data-driven solutions such as deep learning show promising classification accuracy, their lack of interpretability restricts their usage in clinics [14,15,16,17]. Moreover, previous research has ignored spatial position information, despite the variations in the closeness and distribution between staining regions in different grades’ images, indicating the potential use of the topological theory in CFS evaluation. To the best of our knowledge, the topological features have not been applied in corneal-staining evaluation.

Therefore, we considered whether topological attributes might uncover a more efficient underlying differentiating mechanism between different staining grades. We proposed an automated machine learning-based method for evaluating corneal fluorescein staining in dry-eye disease, which includes a set of multiscale topological features designed to capture spatial connectivity and distribution information between staining regions.

## 2. Materials and Methods

### 2.1. Data Set

The retrospective, single-center research was conducted at Beijing Tongren Hospital, Capital Medical University. Patients diagnosed with dry eye based on the Dry Eye Workshop II criteria between 2021 to 2022 were included [19]. Patients with a history of ocular injury or infection, contact lens wear within the last 6 months, surgery within the previous 12 months, or systemic autoimmune diseases such as Sjogren’s syndrome and systemic lupus erythematosus were excluded. The study adhered to the principles of the Declaration of Helsinki. Written informed consent was obtained from all participants.

All corneal images were acquired by a single technician using a photo slit-lamp system (BX 900, Haag-Streit, Bern, Switzerland) with 10× magnification. The cornea was stained with fluorescein by introducing a wet fluorescein sodium ophthalmic strip (Tianjin Jingming New Technological Development Co., Tianjin, China) into the inferior fornix, and participants were instructed to blink [19]. The camera settings were constant for all participants, and images of the entire cornea were obtained using the blue filter and the barrier yellow filter to observe corneal staining.

### 2.2. Investigator Grading System

Two experienced ophthalmologists assessed corneal staining images independently. The results obtained by the two ophthalmologists were compared and discrepancies were resolved with another ophthalmologist. In dry-eye disease, the inferior zone typically presents the most severe grade of corneal staining. Compared to other areas, the inferior zone has a higher density of staining and provides more features [20]. We assumed that the center of the cornea was located at the middle of pupil, and the distance from the center to the bottom edge of the cornea was regarded as the radius. Therefore, the region of interest (ROI) was located at the lower sector areas, with an included angle of 90°. The ROI in the inferior cornea was finally graded according to the OSS scale. The OSS score was calculated as follows: 0, no staining; 1, one to five staining dots; 2, six to 30 staining dots; 3, more than 30 staining dots [7]. An additional point was added if:(1) there were one or more patches of confluent staining, (2) there were one or more filaments, or (3) staining occurred in the central cornea. As the ROI was the inferior zone of the cornea, the maximum score for each ROI was 5 in our study. The final OSS score was regarded as the true label.

### 2.3. Staining Region Analysis

**Figure 1:**
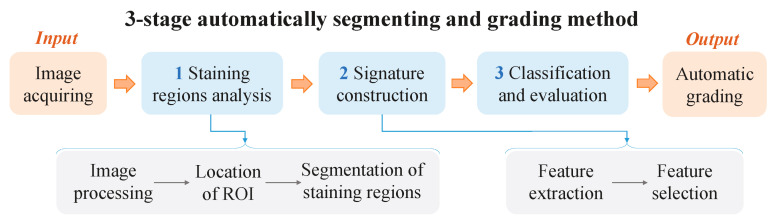
Overview of our proposed automatic segmenting and grading method for corneal fluorescein staining evaluation.

As shown in Figure 1, the stage of staining region analysis was developed to extract the regions of staining. The extraction process consisted of three primary steps: image preprocessing, identification of the region of interest (ROI) in the cornea, and segmentation of staining regions within the ROI. The precise determination of both the ROI and staining regions was critical for achieving optimal performance of the automated grading system.

#### 2.3.1. Image Preprocessing

**Figure 2:**
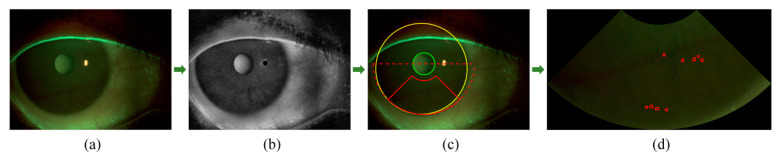
Representative result of image digital analysis: (**a**) original image; (**b**) image after preprocessing; (**c**) location of the region of interest (ROI) (red lines), with the cornea in yellow lines, the pupil in green lines and the semicircle applied to determine ROI in red dashed lines; (**d**) segmentation of staining regions in the ROI (red lines).

To improve the clarity of corneal edges and eliminate reflective areas, we applied a series of image preprocessing operations before determining the ROI. In cases where low-contrast images were obtained, likely due to variations in the light environment during image capture, we employed image enhancement methods to increase the visibility of corneal edges. First, we extracted the red channel from the input color image to enhance the edges and minimize the possible impact of staining (Figure 2a). We then utilized median filtering and contrast-limited adaptive histogram equalization (CLAHE) to decrease noise and improve image contrast [11,21], respectively. Additionally, to address potential reflective regions in the images, Otsu thresholding was applied to detect and eliminate the brightest areas (Figure 2b) [22].

#### 2.3.2. Location of the ROI

Following the preprocessing procedures, we identified the ROI by first detecting the cornea and the pupil. We used Otsu thresholding to binarize and segment the sclera areas, which were outside and brighter than the cornea. We then determined the center and radius of the cornea by identifying the largest diameter in the inner edges of the binarized sclera, corresponding to the circular edges of the cornea. Daugman’s integrodifferential operator was subsequently used to detect the pupil within the cornea [23]. Specifically, we searched for the largest outward radial gradient of the grey intensity of the circular ring under any proposed combination of the center (x0, y0) and radius r, consisting of the circle of the pupil. Daugman’s equation, as presented in Equation (1), was used to compute the pupil’s position. Once the cornea and the pupil were detected, the ROI’s location was determined as the lower sector area with an included angle of 90°. Figure 2c shows the detected cornea, pupil, and ROI.

(1) $$\underset{\left(r,x_{0},y_{0}\right)}{max}|G_{\sigma }\left(r\right)\times \frac{\partial }{\partial r}\int_{\begin{array}{c} r,x_{0},y_{0} \end{array}}\frac{I(x,y)}{2\pi r}ds|$$

#### 2.3.3. Segmentation of Staining Regions in the ROI

To segment the staining regions of the dots or patches in the located ROI image, we employed the top-hat by reconstruction operation in morphology [24]. Specifically, we first extracted the green channel to highlight the green staining regions and then performed an open operation to eliminate small bright details. Next, the ROI image was reconstructed in grayscale using the result from the open operation as a template, which effectively removed the highlights while preserving the background. Finally, the staining regions were segmented by taking the difference between the ROI and the reconstructed result (Figure 2d). Compared with the commonly used threshold segmentation methods, such as Otsu, the top-hat by reconstruction operation showed superior detection performance to staining regions under different contrasts.

### 2.4. Signature Construction

After segmenting the desired stained regions, we proceeded with signature construction, which involved feature extraction and selection to meet the requirements of the classification model. In this stage, we extracted conventional radiomic features, including textural and morphological features. Additionally, we developed a set of multiscale topological features to uncover spatial connectivity and distribution information between staining regions [25]. These additional features aimed to capture the intricate relationships and patterns among the stained regions.

#### 2.4.1. Feature Extraction

Radiomic features, encompassing both textural and morphological characteristics, were calculated, as the previous study mentioned [26,27]. A total of 837 texture features were extracted to analyze the background grayscale textural and pattern information of ROI images. Specifically, this included 18 first-order histogram statistical features and 75 second-order gray matrix features, such as autocorrelation and cluster prominence in the gray-level co-occurrence matrix (GLCM), the gray-level non-uniformity and gray-level non-uniformity normalized in gray-level size zone matrix (GLSZM), the gray-level variance and high gray-level run emphasis in gray-level run length matrix (GLRLM), and so on. These features were calculated in nine forms of images, respectively [26]. In addition to the original image, three image transformation operations were performed to enrich the textural information of the ROI. These operations included Laplacian of Gaussian filtering (LoG) (σ = 1, 2, 3), four components of wavelet decomposition, and local binary patterns (LBPs). Furthermore, to avoid ignoring the information from staining regions, nine morphological features were extracted by calculating the shape characteristics, such as circularity and the area of each segmented staining region [10].

**Figure 3:**
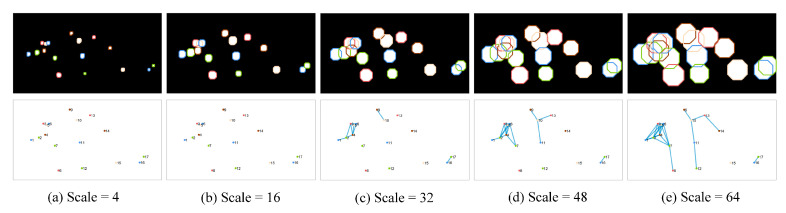
Representative process of constructing topological graphs. The scales shown from column (**a**) to column (**e**) are 4, 16, 32, 48, and 64. Top row: the results of dilation on the staining regions at different scales; bottom row: the results of the constructed topological graph at corresponding scales.

To capture the spatial connectivity and distribution information of staining regions, a set of multiscale graph-theoretic topological features were developed. These features aimed to address characteristics like regional clustered distribution resulting from confluent staining, which cannot be accurately described by textural and morphological features alone [25]. The approach involved considering the centroid of each staining region as the vertex in a graph and estimating the connectivity between staining regions using dilation operations at multiple scales. When two staining regions overlapped after dilation, their nodes were connected by an edge, thus generating a topological graph that reflected the connectivity. As the scale of dilation increased from small to large, the number of edges and the connectivity of the graph exhibited a positive correlation. The construction process of the topological graph is shown in Figure 3. Eight topological features, including vertex degree and eccentricity, were calculated at each scale. These calculations were performed using different disk dilation operators, ranging in size from 4 to 64. A total of 128 multiscale topological features were extracted in 16 scales. The texture features we extracted were the same as those in the literature, and the morphological and topological features extracted are shown in Table 1 [25].

#### 2.4.2. Feature Selection

In order to handle the large number of features (974 normalized features) obtained after calculating all three kinds of features, a four-step feature selection method was employed for signature construction. This approach aimed to reduce the dimensionality of the features set and prevent overfitting of the model without compromising its performance. One-way analysis of variance (ANOVA) and a Pearson redundancy-based filter (PRBF) were applied to eliminate redundant terms in the feature vector [28]. Subsequently, a backward feature selection method based on the linear regression model was used to remove non-significant features [29]. The final selection for important features was determined using a decision-tree method [30], where the importance of each feature was measured by the gini impurity at each node. Higher importance indicated stronger separability of the feature across different classes of the model. Multiple alternative signatures were constructed, based on different combinations of the importance ranking and feature categories.

### 2.5. Classification and Evaluation

The developed machine learning-based model aimed to classify corneal staining images into different grades, based on selected features. To determine the optimal approach for our auto-grading method and evaluate the effect of different categories of features, we extensively explored the performance of different combinations of signatures and models. Specifically, four groups of features were applied in the same support vector machine (SVM) model in order to determine the one that was associated with best performance and accept it as the final signature. Additionally, different combinations of topological and non-topological features (textural and morphological) were investigated to evaluate the contributions of topological features in the classification process. This included the overall top-10 important features, the top-10 important non-topological features, and the top-5 important topological features and non-topological features, respectively. These resulting signatures were then accessed using five kinds of machine learning-based classification models, namely, SVM, Naive Bayes (NB), Decision Tree (DT), Boosting Tree (BT), K-nearest neighbors (KNN), and Random Forest (RF). In our experiments, the original dataset was divided into a training dataset (307 images) and a testing dataset (77 images), in an 8:2 ratio. A ten-fold cross-validation method was used to train and validate each model on the training dataset, while the classification performance of the model was evaluated with the testing dataset.

### 2.6. Statistical Analysis

An intraclass correlation coefficient (ICC) was calculated to assess the interobserver repeatability between two resident ophthalmologists; ICC ≥ 0.8 indicated good reliability. Accuracy (ACC), sensitivity (SEN), precision (PRE), specificity (SPE), F-measure of the confusion matrix, and the area under the receiver operating characteristic (ROC) curve (AUC) were calculated to evaluate the performance of the proposed method. The confusion matrix showed the predicted label and true label of each class, and the ACC was the ratio of the total number of correctly classified samples to the total number of samples. Due to the imbalance between different classes in the dataset, the F-measure was also used to evaluate the harmonic mean of precision and sensitivity. The ROC is a function of sensitivity and specificity, which was established by changing the threshold of the classifier. Generally, AUC was used to evaluate the accuracy of the classification model. The larger the AUC, the better the performance. Kendall’s tau-b correlation coefficient (r_K_) was used to evaluate the correlation between selected features and true labels. All experiments were two-sided tests; *p* < 0.05 was considered to be statistically significant and *p* < 0.01 was considered to be statistically extremely significant.

### 2.7. Implementation Details

The detection and classification experiments in this study were conducted on a personal computer with an Intel Core i9-9900K, 3.60 GHz, and 16 cores. The programming language used for the implemented algorithms was MATLAB software (version R2022a, RRID:SCR_001622). All input images were standardized to a size of 1944 × 2592, and the extracted ROI size was normalized to 596 × 1104. In the preprocessing phase, median filtering was applied with a neighborhood size of 21 × 21. For pupil detection using Daugman’s integrodifferential operator, the preselected range for the pupil center (x0, y0) was a rectangle with the corneal center as the center and 0.35 times the corneal radius as the length. The preselected range for the pupil radius (r) was from 0.15 times to 0.35 times the length of the corneal radius. The calculation angle range for the integrodifferential operator was from 0 degrees to 360 degrees. In the reconstruction of the top-hat operation, a circular structural element with a radius of 10 was used. For machine learning model training, the dataset was divided into a training set and a test set in an 8:2 ratio. Model parameters were optimized using the training set, and the model’s performance was evaluated using the test set. For the SVM model, the kernel function was a Gaussian kernel. The DT model had a maximum split criterion of 4 at each node. The boosting tree model underwent 30 training cycles. The KNN model had a neighborhood size of 10.

## 3. Results

### 3.1. Analysis of Dataset and Subjective Grades

A total of 421 images were acquired and 382 images were enrolled in the study, excluding blurred or poor-exposure images. No image was observed with filament, allowing for the implementation of a five-category model ranging from 0 to 4. There were 41 images (10.7%) with grade 0; 57 images (14.9%) with grade 1; 112 images (29.3%) with grade 2; 59 images (15.5%) with grade 3; and 113 images (29.6%) with grade 4. The median score was 2 (range, 0–4). The ICC values between the two ophthalmologists were 0.789 (95% confidence interval: 0.562–0.881, *p* < 0.001).

### 3.2. Selection and Evaluate of Features

**Figure 4:**
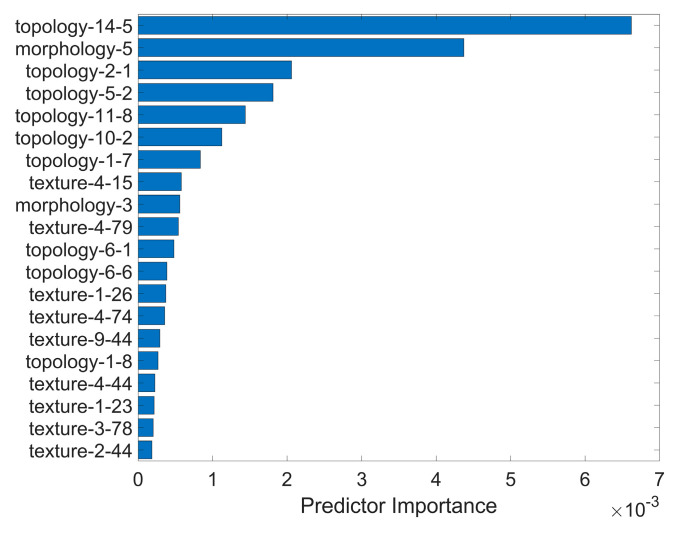
The top-20 importance ranking list for the selected features.

In this study, 992 initial features were extracted from the ROI and the staining regions of each image. These included 837 textural features, 9 morphological features, and 128 topological features. Specifically, texture features were directly computed within the localized ROI image, while morphological and topological features involved operations on the detected stained regions. Morphological features calculated information for each connected component of the stained regions, while topological features were evaluated from the graph structure constructed from the overall results of the stained region detection. During the process of feature selection, ANOVA retained 556 features, PRBF preserved 199 features, and. finally, backward feature selection based on a linear regression model yielded 44 features. Additionally, the decision-tree feature selection method predicted the importance of features in classification. The top-20 important features are shown in Figure 4, demonstrating that six out of the top seven ranked features were topological features.

The top-20 important features are shown in Figure 4. We evaluated the performance of four groups of features with an SVM model on the training (10 FCV) and testing datasets, respectively. The classification results are summarized in Table 2. The overall top-10 important features provided the best performance, with an ACC of 82.67% and an AUC of 96.59%. These results were significantly higher than those of the other feature groups. Similarly, the topological top-5 important features exhibited superior performance (ACC: 74.67%, AUC: 95.36%) compared to the non-topological top-5 (ACC: 64.00%, AUC: 84.14%). This finding suggests that topological features made a more significant contribution than other types of features.

ACC, accuracy; SEN, sensitivity; PRE, precision; SPE, specificity; F-M, F-measure; AUC, area under the receiver operating characteristic curve.

**Figure 5:**
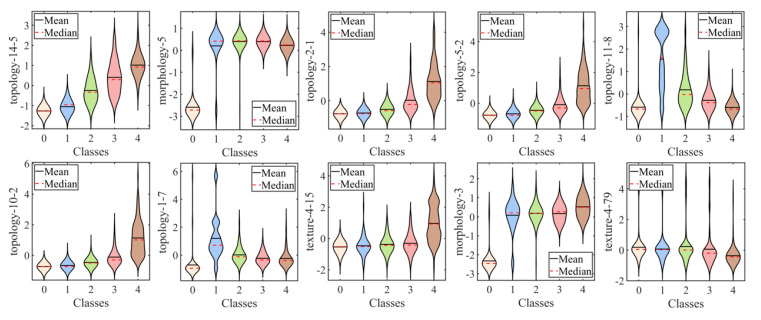
The violin plots of the top-10 important features.

The overall top-10 important features consisted of two textural, two morphological, and six topological features. Their explanations are shown in Table 3. For more detail, the distribution of each feature in the five classes is presented in Figure 5. The correlation between features and true labels is shown in Table 4. All topological features except “topology 11-8” and “topology 1-7” were strongly correlated to true labels (r_K_ > 0.7, *p* < 0.001).

Since some features in score 0 images had zero values, which may interfere with the potential correlation, the result of removing the score 0 images is also shown in Table 4. Please note that “topology-11-8” showed a negative relationship (r_K_ = −0.6, *p* < 0.001).

### 3.3. Effectiveness of Selected Features among Different Classification Models

**Figure 6:**
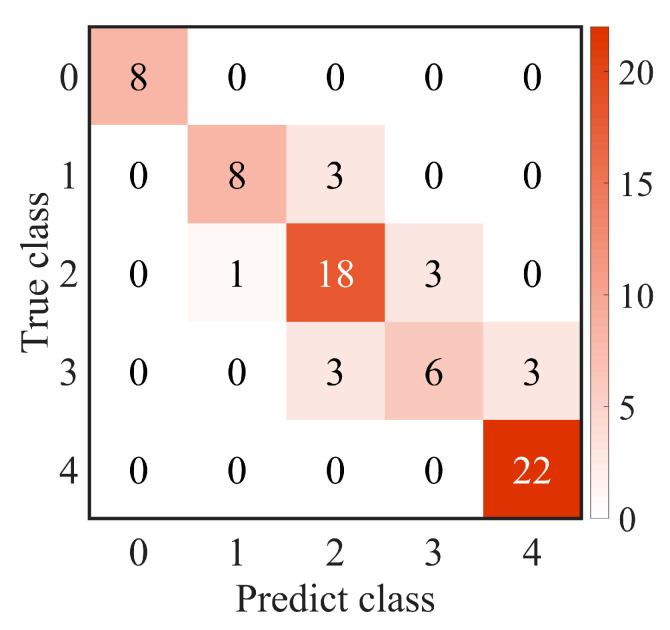
The confusion matrix with the top-10 important features in support vector machines (SVMs).

**Figure 7:**
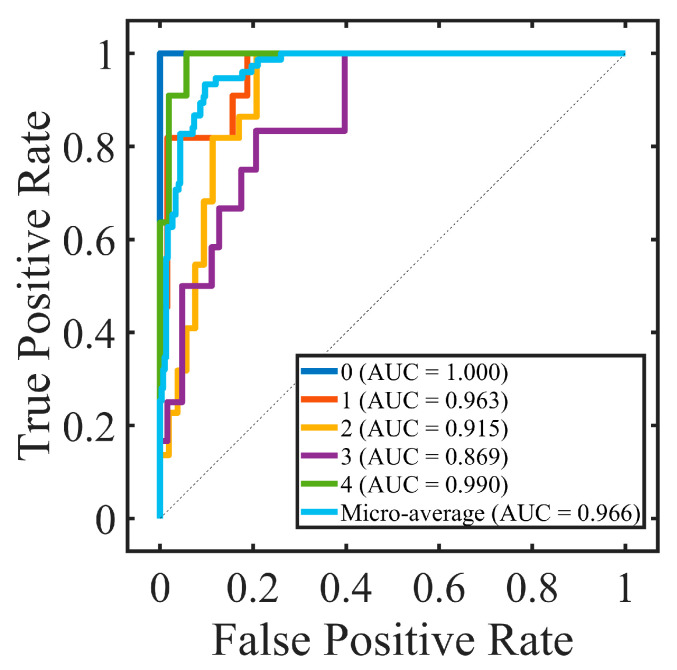
The results of receiver operating characteristic (ROC) and area under the curve (AUC) with the top-10 important features in support vector machines (SVMs) for each class. The performance metrics for the average ROC curve were computed using the micro-averaging method.

The effectiveness of the selected three groups of features was evaluated using five different machine learning-based classification models (SVM, DT, BT, NB, KNN, and RF) to assess their generalization performance. As shown in Table 5, among the three feature groups, the overall top-10 important features consistently achieved the highest performance across all classification models. The topological top-5 features outperformed the non-topological features within the same range, indicating a robust effectiveness of the selected topological features. Moreover, SVM showed the best performance compared with other models (ACC: 82.67%, AUC: 96.59%). To further analyze the grading process, the confusion matrix and ROC curve are presented in Figure 6 and Figure 7, respectively. The confusion matrix highlights excellent performance in distinguishing class 0 and class 4, while some errors were observed between class 3 and class 2, as well as class 3 and class 4.

## 4. Discussion

In this study, we developed an automatic machine learning-based method for corneal fluorescein staining evaluation. Our findings highlighted the significance of topological features as clinical characteristics in the evaluation of corneal fluorescein staining in dry-eye disease. This end-to-end model consisted of segmenting staining regions, extracting their radiomic characteristics, and differentiating them into the corresponding grades. The resulting radiomic signature was constructed from a combination of multiscale topological features, as well as textural and morphological features. The experimental results of our clinical dataset demonstrated the good performance of the proposed model with ACC 82.67% and AUC 95.36%. Moreover, we identified the 10 most important interpretable image-based features, with topological features playing a crucial role. These topological features captured the spatial connectivity and distribution information among staining regions, thereby holding promising in guiding clinical decision-making for the diagnosis and treatment of dry-eye diseases.

The key component in our proposed method was the usage of multiscale topological features that scrutinized the spatial connectivity and distribution among staining regions. These features significantly contributed to the discriminatory ability of the model. This is evident in Figure 4, where the top-ranked radiomic features after selection include six topological attributes among the top seven characteristics. Additionally, the superior performance of topological features compared to non-topological features was consistently observed across different machine learning-based classification models, as shown in Table 5. This indicated the effectiveness of topological features in distinguishing between different grades of corneal staining. Moreover, Table 4 reveals a significant positive or negative correlation between topological characteristics and subjective gradings, which was not observed in other textural and morphological features. These findings underscore the unique contribution of topological features in capturing relevant spatial information related to corneal staining.

The observation of an increasing diameter of scale 56 (“topology-14-5”) with higher true labels suggested a linear distribution of fluorescein staining. In the context of clinical practice, we interpreted this linear distribution as being horizontal. The horizontal distribution is associated with incomplete blinking, which refers to a blink cycle where the upper lid turns to the upstroke before making contact with the lower lid [31,32]. Incomplete blinking is commonly observed in dry-eye patients [33]. Incomplete blinks extend the period of horizontal strip area exposure to desiccating stress, leading to more corneal epithelium defects in that region [34]. Therefore, the presence of a linear distribution of staining may serve as an indicator of incomplete blinking and the associated increased risk of corneal epithelial damage. Other topological features revealed the aggregation of corneal staining in the inferior zone, which has rarely been reported in previous studies. The number of subgraphs of scale 8 (“topology-2-1”) and the average vertex degree of scale 20 (“topology-5-2”) and 40 (“topology-10-2”) positively correlated with the true label, which indicated a tendency for staining to cluster. The aggregation phenomenon may be attributed to the main mechanism of corneal epithelium cell damage in DED, which is tear hyperosmolarity. It has been proven that hyperosmolarity can be localized [3]. Additionally, tear film is more likely to break up in areas in damaged epithelium, leading to an increase in osmolarity in those regions. This creates a vicious cycle, resulting in more corneal epithelium defects in the same area. Furthermore, the more staining in the fixed area (the inferior zone of the cornea) inevitably affects the value of such topological features. These topological features highlight the crucial role they play in distinguishing the severity of corneal staining and provide valuable insights into the spatial connectivity and distribution of staining regions in a clinical setting.

We extensively explored different combinations of feature signatures and models to identify the approach that yielded the best performance for our computer-aided diagnosis method. The selection of the support vector machine (SVM) model as the best-performing classification model in our study was based on its high accuracy (ACC) of 82.67% and area under the receiver operating characteristic curve (AUC) of 96.59%. The high ACC and AUC values obtained with the SVM model indicated its effectiveness in accurately classifying corneal staining images into different grades.

There were some previous studies that reported similar results in staining-regions evaluation. While some studies focused on semi-automatic methods for locating the region of interest (ROI) in the cornea [9,10], our method was fully automated, providing accurate localization and segmentation of staining regions. Additionally, previous studies mainly relied on image processing or deep-learning techniques for corneal region segmentation, without specifically analyzing the spatial connectivity and distribution of staining regions [11,12,18]. In contrast to approaches that only consider parameters such as the number and area of staining regions, we introduced topological features to capture the spatial characteristics and the confluence of staining regions. This provided a more comprehensive analysis of corneal staining patterns. By incorporating these topological features along with textural and morphological traits, our machine learning-based classification model demonstrated improved performance in grading corneal staining.

The effectiveness of the topological features identified in our study holds promise for clinicians in diagnosing dry-eye disease. The integration of topological features with other radiomic characteristics enhances the classification performance and provides valuable insights into the spatial characteristics of corneal staining. This information can, potentially, aid clinicians in diagnosing and managing dry-eye disease more effectively.

There were several limitations in our study that should be acknowledged. First, the segmentation accuracy of large staining patches could be improved. Due to the low contrast and ambiguous edges of the patches, the segmentation accuracy was compromised. Future studies could explore more efficient methods, such as deep learning, for accurately locating the ROI and segmenting staining regions. Second, the performance of the proposed method should be further validated on a larger dataset. Although our study included a substantial number of images, a larger dataset would provide more robust evidence of the effectiveness of the proposed method. Lastly, the classification performance for score 3 was relatively insufficient. It is possible that score 3 included two distinct conditions: true score 3 and score 2 with an additional score for confluent staining. This may have resulted in a misclassification as score 4 or score 2. Future research could focus on designing a more refined classification model to improve the differentiation of score 3 from other grades. Addressing these limitations in future studies will help to further enhance the accuracy and applicability of the proposed method in corneal fluorescein staining evaluation.

## 5. Conclusions

Our study demonstrated the effectiveness of a fully automatic machine learning-based method for corneal fluorescein staining analysis. The integration of topological features with other radiomic characteristics enhanced the classification performance and provided valuable insights into the spatial characteristics of corneal staining. This information can, potentially, aid clinicians in diagnosing and managing dry-eye disease more effectively.

## Figures and Tables

**Table 1:** Extracted topological features.

Feature Categories	Feature Names
Topological features	Number of subgraphs Average vertex degree Maximum vertex degree Average vertex eccentricity Diameter Average clustering coefficient Giant connected component ratio Percentage of isolated points
Morphological features	Mean area Total area Mean perimeter to area ratio Total perimeter to area ratio Mean circularity Mean perimeter Total perimeter Minimum external rectangle aspect ratio Number

**Table 2:** Classification results of different groups of features in SVM.

Feature Groups	ACC	SEN	PRE	SPE	F-M	AUC
The overall top-10 important features	82.67	83.71	80.91	80.91	81.80	96.59
The top-10 important non-topological features	64.00	53.53	57.88	57.88	54.22	90.64
The top-5 important topological features	74.67	65.82	70.91	70.91	67.60	95.36
The top-5 important non-topological features	64.00	46.48	56.36	56.36	49.90	86.14

**Table 3:** Explanation of the top-10 important features.

Feature Names	Feature Explainations
Topology-14-5	The diameter in topological features of scale 56
Morphology-5	The mean circularity in morphological features
Topology-2-1	The number of subgraphs in topological features of scale 8
Topology-5-2	The average vertex degree in topological features of scale 20
Topology-11-8	The percentage of isolated points in topological features of scale 44
Topology-10-2	The average vertex degree in topological features of scale 40
Topology-1-7	The giant connected component ratio in topological features of scale 4
Texture-4-15	The skewness in textural features of LOG transform of sigma 3
Morphology-3	The mean area perimeter ratio in morphological features
Texture-4-79	The large area low gray-level emphasis in textural features of GLSZM of LOG transform of σ = 3

**Table 4:** The correlation between the top-10 important features and true labels.

Feature Names	OSS 0 to 4		OSS 1 to 4	
	r_K_	*p* Value	r_K_	*p* Value
Topology-14-5	0.74	<0.001 *	0.68	<0.001 *
Morphology-5	−0.02	0.694	−0.30	<0.001 *
Topology-2-1	0.74	<0.001 *	0.71	<0.001 *
Topology-5-2	0.72	<0.001 *	0.68	<0.001 *
Topology-11-8	−0.31	<0.001 *	−0.60	<0.001 *
Topology-10-2	0.75	<0.001 *	0.71	<0.001 *
Topology-1-7	−0.10	0.006	−0.39	<0.001 *
Texture-4-15	0.39	<0.001 *	0.40	<0.001 *
Morphology-3	0.35	<0.001 *	0.18	<0.001 *
Texture-4-79	−0.31	<0.001 *	−0.31	<0.001 *

OSS, the Sjögren’s International Collaborative Clinical Alliance Ocular Staining score; r_K_, Kendall’s tau-b correlation coefficient; *, *p* value < 0.05.

**Table 5:** Classification results for different groups of features in SVM, DT, BT, NB, and KNN.

Models	Feature Groups	ACC	SEN	PRE	SPE	F-M	AUC
SVM	A-10	82.67	83.71	80.91	80.91	81.80	96.59
	T-5	74.67	65.82	70.91	70.91	67.60	95.36
	NT-5	64.00	46.48	56.36	56.36	49.90	86.14
DT	A-10	73.33	78.40	71.97	71.97	73.66	90.67
	T-5	69.33	62.55	65.45	65.45	62.60	87.79
	NT-5	57.33	41.68	50.23	50.23	44.58	84.97
BT	A-10	74.67	75.77	74.55	74.55	74.88	93.25
	T-5	69.33	68.08	68.03	68.03	67.57	91.48
	NT-5	68.00	60.02	59.17	59.17	56.39	87.62
NB	A-10	76.00	78.50	76.21	76.21	76.78	88.35
	T-5	68.00	71.24	67.58	67.58	68.53	85.41
	NT-5	62.67	57.72	56.14	56.14	55.10	88.19
KNN	A-10	80.00	79.66	78.18	78.18	78.26	95.44
	T-5	74.67	74.88	73.18	73.18	73.59	91.43
	NT-5	60.00	55.72	55.23	55.23	55.05	86.40
RF	A-10	77.33	78.52	78.03	78.03	78.13	94.86
	T-5	72.00	72.32	70.61	70.61	70.37	90.96
	NT-5	68.00	65.94	64.17	64.17	64.73	90.28

A-10, the overall top-10 important features; T-5, the top-5 important topological features; NT-5, the top-5 important non-topological features; ACC, accuracy; SEN, sensitivity; PRE, precision; SPE, specificity; F-M, F-measure; AUC, area under the receiver operating characteristic curve; SVM, support vector machine; DT, Decision Tree; BT, Boosting Tree; NB, Naïve Bayes; KNN, K-Nearest Neighbor, RF, Random Forest.

## Data Availability

The data presented in this study are available on request from the corresponding author, upon reasonable request.
